# Hereditary, non HINT1 related, axonal neuropathy with neuromyotonia

**DOI:** 10.1007/s10072-025-08022-z

**Published:** 2025-02-26

**Authors:** Kanellos C. Spiliopoulos, Dimitra Veltsista, Eirini Veltsou, Valentini Tzimogianni, Go Hun Seo, JiHye Kim, Zoi Lygerou, Elisabeth Chroni

**Affiliations:** 1 Department of Neurology, School of Medicine, University of Patras, Patras, Greece; 2 Molecular Genetics Unit, Department of Gen. Biology, School of Medicine, University of Patras, Patras, Greece; 3 3billion Inc, Seoul, South Korea

**Keywords:** Neuromyotonia, Charcot-Marie-tooth, Myelin protein zero, Axonal neuropathy, Neuromuscular ultrasound

## Abstract

**Background:**

To date, neuromyotonia in the context of an inherited axonal neuropathy has been linked to autosomal recessive mutations in the histidine triad nucleotide binding protein 1 (HINT1) gene. In this study we describe two unrelated male patients with late-onset, predominantly motor, axonal neuropathy with neuromyotonia, who carried an autosomal dominant c.103G > A mutation in the myelin protein zero (*MPZ*) gene (NM_000530.8:c.103G > A, p.Asp35Asn), identified by whole-exome sequence analysis (WES).

**Case Descriptions:**

The first patient presented progressive leg muscle weakness and stiffness with difficulty in walking, pain and increased creatine kinase levels,during his fifth decade of life. Electrophysiological examination revealed findings of an axonal, length-dependent polyneuropathy with spontaneous activity, mainly neuromyotonia. Over the 20-year disease course since the first reported symptoms, muscle weakness gradually worsened and he is currently unable to walk without assistance. A second male patient, unrelated to the first one, showed similar clinical and electrophysiological features of a length-dependent axonal neuropathy with neuromyotonia. WES detected the same MPZ missensevariant.

**Conclusion:**

This study suggests a novel entity in the spectrum of Charcot-Marie-Tooth hereditary neuropathies, characterized by autosomal dominant axonal neuropathy with neuromyotonia (AD-NMAN).

**Supplementary Information:**

The online version contains supplementary material available at 10.1007/s10072-025-08022-z.

## Introduction

Neuromyotonia refers to a peripheral nerve hyperexcitability condition that causes muscle stiffness and twitching and is electrophysiologically characterized by spontaneous, continuous motor unit discharges of high firing frequencies (150–300 Hz) [1]. Although acquired neuromyotonia is a well-known entity commonly of an autoimmune origin, this phenomenon in association with hereditary neuropathies is restricted to autosomal recessive, axonal neuropathy (AR-NMAN) which is typically caused by mutations in the histidine triad nucleotide binding protein 1 (HINT1) gene [1, 2].

Herein, we report two male patients with an adult-onset axonal neuropathy and neuromyotonia, diagnosed with a myelin protein zero (MPZ) neuropathy due to an autosomal dominant c.103G > A mutation. Mutations in the MPZ gene, the encoding gene of the basic glycoprotein component of the myelin sheath, are responsible for a spectrum of hereditary neuropathies, including both demyelinating and axonal forms [3].

## Case 1

A 62-year-old male was referred to our Neuromuscular Disease Department for re-evaluation due to progressive muscle weakness and atrophy of the lower and upper limbs. Until the age of 42, the patient had not experienced any specific difficulty in his daily activities as a farmer. At the age of 43, he first noticed pain in the distal parts of the upper and lower limbs and also experienced frequent cramping, continuous contractions and progressive muscle weakness in the lower limbs with difficulty in walking. Increased creatine kinase (CK) levels (ranging between 200 and 1000 IU/L) raised the suspicion of a neuromuscular disease. His family history was negative for neuromuscular disorders. The initial needle electromyographic examination revealed neuromyotonic discharges raising the suspicion of an autoimmune peripheral nerve hyperexcitability syndrome. The patient was unsuccessfully treated with monthly intravenous immunoglobulin infusions for 2 years.

When first examined in our department at the age of 49 years, we observed spontaneous irregular muscle twitching in lower limb muscles and high-arch feet with toe clawing. The muscle strength was normal in upper limbs and reduced in lower limbs (thigh muscles 4/5, leg and foot muscles 3–4/5 on Medical Research Council [MRC] scale). No cranial nerve involvement or pyramidal signs were detected. Pain and vibration sense were reduced up to the ankle, and deep tendon reflexes were absent at the ankles and diminished elsewhere. Autoantibody profile was negative and CK levels remained high (1000 IU/L).

### Electrophysiological findings

**Fig. 1:**
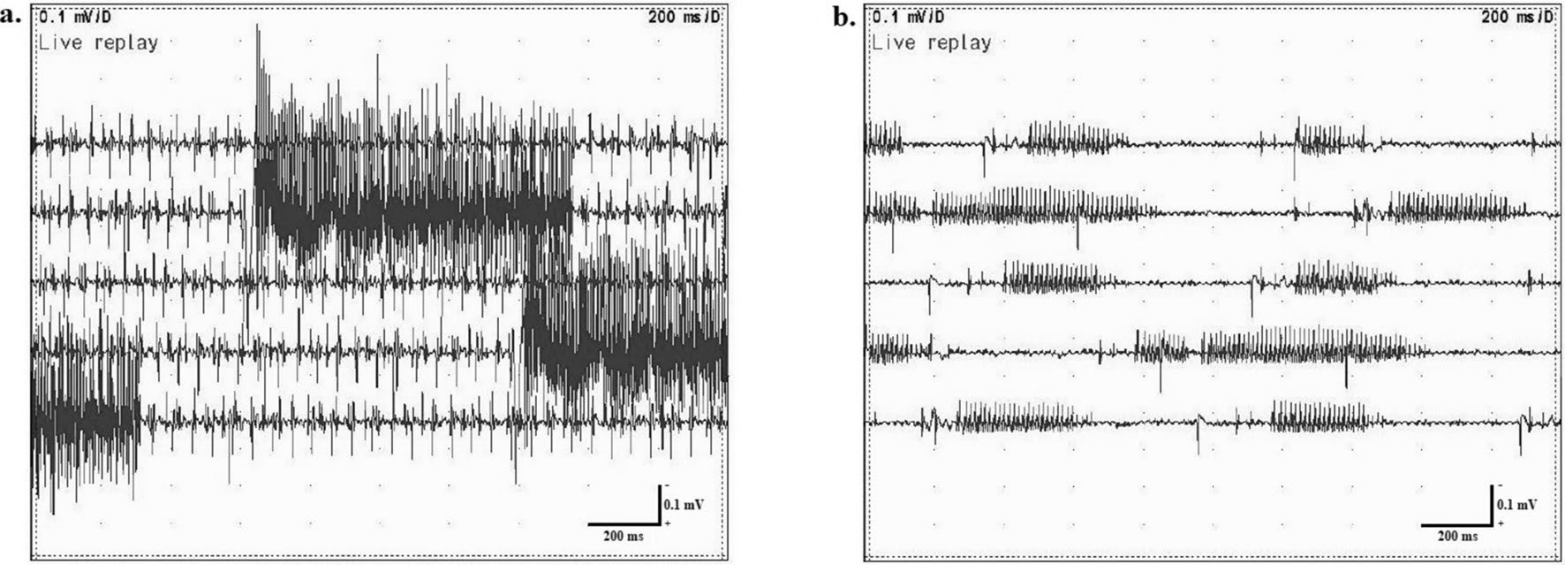
Neuromyotonic discharges of high firing frequency in (**a**) tibialis anterior and (**b**) biceps brachii muscles

Nerve conduction study was indicative of an axonal, predominantly motor, length-dependent polyneuropathy (Table 1). Needle electromyography revealed neuromyotonic and myokymic discharges in all studied muscles (including biceps brachii, rectus femoris and tibialis anterior) (Fig. 1). Examples of the recorded neuromyotonic discharges are shown in Supplementary Video 1.

**Table 1:** Neurophysiological data of the first examined patient

	First visit Patient aged 49 years	Follow-up Patient aged 62 years	Normal limits
**Nerve Conduction Study**			
Motor conduction			
*Ulnar nerve*			
Latency (m/s)	3.3	3.3	≤ 3.5
Amplitude wrist/elbow st. (mV)^a^	5.1/4.7	4.0/4.5	≥ 4.0
Velocity wrist to elbow (m/s)	56.2	53.1	≥ 50
F-waves minimum latency (m/s)	28.7	29.4	< 32
*Peroneal nerve*		Not evocable	
Latency (m/s)	5.1		< 5.5
Amplitude ankle/knee st. (mV)^a^	0.6/0.4		> 2.0
Velocity (m/s)	45.7		> 42
F-waves minimum latency (ms)	Absent		< 58
Sensory conduction			
*Sural nerve*		Not evocable	
Amplitude (mV)^a^	1.7		≥ 7.0
Velocity (m/s)	40		≥ 40

^a^ Measurements baseline to negative peak for motor and peak to peak for sensory responses; compound muscle action potential were recorded from abductor digiti minimi (ulnar) and extensor digitorum brevis (peroneal nerve)

### Genetic analysis

Whole-exome sequencing (WES) was performed in the laboratory of 3 billion Inc., Seoul, South Korea and revealed that the patient carried the following pathogenic missense variant in the MPZ gene, in heterozygocity:

DNA: NM_000530.8:c.103G > A.

Protein: NP_000521.2:p.Asp35Asn.

There was no pathogenic or likely pathogenic variant detected in the protein-coding regions of the HINT1 gene, which were covered well (100% covered at > = 20x).

### Current examination

Over the course of 13 years, the mobility status of the patient has radically changed, since the patient in his recent evaluation was hardly ambulatory requiring bilateral support. Limb muscle strength was further reduced, involving also the upper limbs (elbow muscles and wrist extensors 4/5, thigh muscles 3–4/5, leg and foot muscles 1–2/5 on MRC scale). Sensory deficits were found in pain, touch and vibration elements in a stocking-glove pattern. Neurophysiological examination revealed further deterioration in nerve conduction studies (Table 1), as well as neuromyotonic discharges in needle electromyography of the tibialis anterior muscle. Muscle ultrasound examination showed increased echogenicity of the affected muscles, atrophies, as well as involuntary continuous muscle activity (Suppl. Video 2). Ultrasound-estimated cross sectional areas of median, ulnar and peroneal nerves, throughout their entire course, were within normal range.

## Case 2

A second patient, unrelated to the first one, with the same mutation, came to our attention. Both patients are residents of certain geographical region, living within a radius of 300 km from each other. This is a 67-year-old male with a 12-year history of sensorimotor polyneuropathy starting from the distal lower limbs and gradually progressing to the upper limbs accompanied by frequent episodes of muscle stiffness. Clinical examination showed MRC score of 0–1/5 on ankle dorsi- and plantar flexion, 4/5 in proximal leg muscles and small hand muscles, absent Achilles tendon reflexes bilaterally and suppressed all sensory modalities up to the knee level. Neurophysiology revealed length-dependent axonal polyneuropathy with neuromyotonic discharges in the tibialis anterior muscle. Routine blood testing was unremarkable except for slightly increased CK (ranging from 200 to 300 IU/L) and the investigation for auto-antibodies, paraprotein, and amyloidosis was negative. WES revealed the exact same missense variant in MPZ gene (DNA: NM_000530.8:c.103G > A, Protein: NP_000521.2:p.Asp35Asn) in heterozygocity.

## Discussion

Our patients showed clinical features of a chronic, distal, predominantly motor impairment with gait difficulties and electrophysiological findings of an axonal length-dependent neuropathy with neuromyotonia. Genetic testing revealed an autosomal dominant MPZ c.103G > A mutation, categorized in the Charcot-Marie-Tooth (CMT) 2 type, which, to our knowledge, are the first reported cases of hereditary neuropathy with neuromyotonia, not related to a mutation in HINT1.

More than 200 different pathogenic MPZ mutations contribute to the heterogeneity in genotype-phenotype correlations [2]. MPZ neuropathies could be categorized in various CMT phenotypes, including congenital hypomyelination neuropathy, Dejerine-Sottas syndrome as well as the demyelinating CMT1b and axonal CMT2I/J types. Despite sharing the common features of progressive muscle weakness, foot deformities and length-dependent sensory impairment, CMT1b is characterized by the early-onset at childhood, whereas CMT2I/J has an onset at adult life [2, 3].

The MPZ c.103G > A mutation, causing an Asp35Asn amino acid substitution, has previously been described in 3 patients with a late-onset axonal CMT2 neuropathy [4]. Similarly to our cases, muscle weakness predominantly involved the lower limbs, whereas the disease onset varied between the 3rd and 7th decade. Neuropathy in our study was typically axonal; there were no conduction slowing or increased nerve cross-sectional area in ultrasound testing as expected in demyelinating neuropathies.

The disease, despite the late onset, may progress rapidly, as observed in the first patient, who, after a 20 year-course, is currently in need of bilateral walking support. This finding is consistent with longitudinal assessments in the literature showing a more rapid progression in axonal forms of MPZ neuropathies than in demyelinating forms [5]. The natural history of the MPZ axonal type neuropathy could be attributed to disrupted Schwann cell-axon interactions and dysfunction in MPZ-mediated signal transduction [5, 6]. Given the role of axo-glial interactions in stabilizing nodes and paranodes, one could hypothesize that certain CMT2-related MPZ mutations, like in our patients, could cause nerve membrane hyperexcitability which leads to the phenomenon of neuromyotonia [1, 6].

The increased CK levels observed in both patients could imply an additional muscle component in the MPZ related syndrome. In a recent study, muscle biopsy in patients with MPZ-gene mutations and either CMT1 or CMT2 phenotypes showed alterations indicative of mitochondrial myopathy, of unexplained mechanism [6]. However, primary myopathy as the cause of raised CK is unlikely, since MPZ is not expressed in muscles at detectable levels, as documented in the GTEX project (online at https://www.gtexportal.org/). In our cases, the most plausible explanation for CKemia could be the combination of denervation, continuous neuromyotonia and muscle contractions. The link between muscle hyperactivity and muscle injury in the context of hereditary MPZ neuropathy requires validation in future research.

Finally, these two cases highlight the importance of performing genetic testing in patients with apparent acquired inflammatory or autoimmune neuropathies, particularly when refractory to treatment, to avoid the consequences of misdiagnosis and therapy.

## Conclusion

To conclude, the identification of these two cases suggests that the MPZ-related autosomal dominant axonal neuropathy with neuromyotonia (AD-NMAN) could constitute a novel entry in the heterogeneous spectrum of CMT neuropathies.

## Supplementary Information

Below is the link to the electronic supplementary material. 

ESM1 Examples of needle electromyography findings of neuromyotonia in tibialis anterior and biceps brachii. (MP4 23.9 MB) 

ESM2 Ultrasound-detected involuntary activity in the right biceps brachii and anterolateral leg muscles. (MP4 32.1 MB)
